# Histone H3 and H4 acetylation patterns are more dynamic than those of DNA methylation in *Brachypodium distachyon* embryos during seed maturation and germination

**DOI:** 10.1007/s00709-017-1088-x

**Published:** 2017-02-24

**Authors:** Elzbieta Wolny, Agnieszka Braszewska-Zalewska, Daria Kroczek, Robert Hasterok

**Affiliations:** Department of Plant Anatomy and Cytology, Faculty of Biology and Environmental Protection, University of Silesia in Katowice, 28 Jagiellonska Street, 40-032 Katowice, Poland

**Keywords:** *Brachypodium distachyon* embryo, Epigenetic modifications, H4K16ac, H3K18ac, DNA methylation

## Abstract

The transition of seeds from a dry to a metabolically active state requires significant changes in both the spatial and temporal patterns of gene expression, and this transcriptional reprogramming involves various modifications of the chromatin structure. There are several factors that can greatly influence the structure of chromatin, one of which is the chemical modifications of histone proteins and DNA itself. In this study, we analysed the distribution of three epigenetic markers, i.e. acetylation of histone H4 (H4K16ac) and histone H3 (H3K18ac) as well as DNA methylation (5mC) in *Brachypodium distachyon* embryos during the four stages of seed development—maturation, desiccation (quiescence), imbibition and germination. Our results indicate that both H4K16ac and H3K18ac are at a very high level in embryos during seed imbibition, but that the patterns of DNA methylation are considerably more stable in embryos during seed development.

## Introduction

One of the most important stages in the plant life cycle is the period of germination, which begins with imbibition and ends with the emergence of the radicle. Germination causes the embryo transition from a state of quiescence in the dry seed to a state with a highly active metabolism (Bewley 1997; Nonogaki et al. 2010). The uptake of water by a mature dry seed is triphasic. Phase I includes a rapid initial uptake in which the seeds are completely soaked, the enzymes crucial for the initial germination are activated and the various storage compounds that provide nutrition and energy for seed germination begin to be metabolised. Phase II is a plateau phase in which both metabolism and cellular activity increase quickly, and the enzymes that are involved in various physiological processes and morphogenesis are abundantly expressed. In phase III, water uptake increases, plants absorb moisture from the surroundings, cell divisions are intensified and large amounts of stored reserves are mobilised (Bewley 1997; Dong et al. 2015). A critical point during germination is the transition from a dry and quiescent seed to an imbibed and metabolically active one. The switch from one phase to the next requires significant changes in both the spatial and temporal patterns of gene expression. The genes that control the ‘new’ state require activation, whereas the genes that were required for the ‘old’ state must be repressed (van Zanten et al. 2011). This transcriptional reprogramming involves an active modification of the chromatin structure. Gene expression can be influenced by epigenetic modifications such as DNA methylation and various posttranslational modifications of the N-terminal histone tails (Jenuwein and Allis 2001). Many studies have indicated that chromatin organisation is a dynamic process and that it undergoes a considerable rearrangement during the germination of seeds and plant development. Epigenetic modifications of the chromatin structure are crucial for many biological processes and act on many genes during the development and seed germination (Zhang and Ogas 2009).

To date, we have been investigating the epigenetic modifications within embryos at three different physiological stages during seed development in *Brachypodium distachyon* (Brachypodium). In Wolny et al. (2014), we demonstrated that the spatial distribution and abundance of three (H4K5ac, H3K4me1, H3K4me2) epigenetic markers in ‘matured’, ‘dry’ and ‘germinating’ Brachypodium embryos is considerably different in particular organs and tissues and we linked this with the switch of the gene expression profiles in various parts of the developing embryo. However, seed imbibition during the first phase of germination has not been analysed in Brachypodium to date. Hence, three types of epigenetic modifications, i.e. acetylation of histone H4 (H4K16ac) and histone H3 (H3K18ac) and DNA methylation (5mC), which were investigated in embryos at four different stages of seed development, are presented here.

## Material and methods

Four types of *B*. *distachyon* (reference line Bd21) embryos, i.e. matured, ‘dry’ (desiccated), imbibed and germinating, were used in this study. Matured embryos were selected from grains 30 days after fertilisation (DAF); ‘dry’ embryos were derived from dry, 3-month-old seeds. Imbibed and germinating embryos originated from seeds that were placed on moist filter paper in Petri dishes at RT in the dark for 4 and 12 h, respectively. Whole seeds, including the appropriate embryos, were fixed in 4% formaldehyde in PBS for the immunodetection of histone modifications or in 3:1 methanol/glacial acetic acid for the detection of DNA methylation. The procedures of embryo embedding in Steedman’s wax (Steedman 1957) and slide preparation were done according to Wolny et al. (2014). The immunostaining was carried out as described by Braszewska-Zalewska et al. (2010, 2012, 2013). The following antibodies against modified histones and DNA were used: anti-acetyl histone H4 at lysine 16 (1:100; Abcam, Cat. no. 109463), anti-acetyl histone H3 at lysine 18 (1:100; Abcam, Cat. no. 1191) and anti-5mC (Abcam, Cat. no. 10805). As a control, antibodies against unmodified histone H3 (Abcam, Cat. no. 1791) and H4 (Abcam, Cat. no. 10158) were used. The incubation with the primary antibodies was done at 4 °C overnight. Either Alexa Fluor 488 goat anti-rabbit IgG (Invitrogen, Molecular Probes, Cat. no. A-11010) or Alexa Fluor 488 goat anti-mouse IgG (Invitrogen, Molecular Probes, Cat. no. A-11017) was used as the secondary antibody, and the incubation with the secondary antibody was done at 37 °C for 1 h. Slides were mounted in Vectashield (Vector Laboratories) containing 2.5 μg/ml DAPI. Fluorescence of DAPI (excitation 405 nm, emission 425–475 nm) and Alexa 488 (excitation 488 nm, emission 500–600 nm) was registered using an Olympus FV1000 confocal system (Olympus, Poland) equipped with an Olympus IX81 inverted microscope, a ×40 Plan Apo oil-immersion objective lens, a 50 mW diode laser (excitation 405 nm) and a 40 mW multi-line (excitations 457/488/515 nm) argon laser. Image processing operations were executed using an ImageJ Fiji package. For each embryo type, from three to five embryos were sectioned and subjected to the immunodetection procedure. Photomicrographs were taken from three to five sections from middle part of a specific embryo.

The fluorescence intensities of Alexa 488 were measured, as the mean values from integrated density parameter per nuclei using an ImageJ (Fiji). The 8-bit images with Alexa 488 fluorescence were segmented with the threshold value parameter. On average, 300 nuclei were analysed for a single embryo organ/tissue. Data for both the modified histones and the control (unmodified histone H3 and H4) are presented as the mean values from the particular organs/tissues as well as the mean values from the whole embryos at particular stage (Table 1).

**Table 1:** Levels of fluorescence intensities for unmodified histones and their respective modifications

Modification	Organ	Matured	Dry	Imbibed	Germinated
5mC	RAM^a^ SE*	1.39E + 03	1.51E + 03	1.20E + 03	2.00E + 03
		3.56E + 01	6.30E + 01	3.33E + 01	5.24E + 01
	SAM^b^ SE	2.57E + 03	2.28E + 03	2.84E + 03	2.01E + 03
		9.22E + 01	1.18E + 02	6.59E + 01	4.47E + 01
	Scutellum SE	1.92E + 03	1.99E + 03	3.27E + 03	2.38E + 03
		1.58E + 02	1.57E + 02	1.53E + 02	1.39E + 02
	Embryo** SE	1.96E + 03	1.93E + 03	2.44E + 03	2.13E + 03
		9.53E + 01	1.13E + 02	8.40E + 01	7.86E + 01
H3K18ac	RAM SE	1.26E + 03	1.02E + 03	5.20E + 03	1.60E + 03
		2.80E + 01	2.02E + 01	7.66E + 01	2.84E + 01
	SAM SE	1.76E + 03	9.64E + 02	3.97E + 03	1.34E + 03
		3.19E + 01	2.11E + 01	5.44E + 01	2.72E + 01
	Scutellum SE	1.67E + 03	6.21E + 01	4.70E + 03	2.27E + 03
		5.25E + 01	8.47E + 00	2.07E + 02	1.08E + 02
	Embryo SE	1.57E + 03	6.84E + 02	4.62E + 03	1.74E + 03
		3.75E + 01	1.66E + 01	1.13E + 02	5.45E + 01
H4K16ac	RAM SE	1.51E + 02	1.52E + 01	1.86E + 03	1.27E + 03
		7.13E + 00	2.18E + 01	5.03E + 01	6.43E + 01
	SAM SE	1.16E + 03	2.59E + 01	2.45E + 03	7.53E + 02
		2.40E + 01	5.89E + 01	7.47E + 01	1.71E + 01
	Scutellum SE	2.88E + 03	2.77E + 01	3.68E + 03	1.80E + 03
		1.94E + 02	1.88E + 00	1.70E + 02	1.05E + 02
	Embryo SE	1.40E + 03	2.29E + 01	2.66E + 03	1.27E + 03
		7.52E + 01	8.97E + 01	9.84E + 01	6.21E + 01
Unmodified H3	RAM SE	3.12E + 03	2.62E + 03	1.73E + 03	3.11E + 03
		1.17E + 02	6.86E + 01	4.85E + 01	8.70E + 01
	SAM SE	2.11E + 03	1.68E + 03	1.65E + 03	2.46E + 03
		8.36E + 01	4.14E + 01	3.06E + 01	6.63E + 01
	Scutellum SE	2.15E + 03	2.29E + 03	2.25E + 03	2.94E + 03
		1.25E + 02	1.24E + 02	9.49E + 01	1.81E + 02
	Embryo SE	2.46E + 03	2.19E + 03	1.88E + 03	2.84E + 03
		1.09E + 02	7.81E + 01	5.80E + 01	1.11E + 02
Unmodified H4	RAM SE	2.12E + 03	7.74E + 02	1.06E + 03	2.51E + 03
		1.97E + 02	2.77E + 01	3.86E + 01	1.11E + 02
	SAM SE	1.72E + 03	8.71E + 02	7.02E + 02	1.57E + 03
		4.94E + 01	1.50E + 01	1.76E + 01	3.62E + 01
	Scutellum SE	1.44E + 03	1.77E + 03	1.90E + 03	2.00E + 03
		7.76E + 01	1.13E + 02	8.60E + 01	1.18E + 02
	Embryo SE	1.76E + 03	1.14E + 03	1.22E + 03	2.03E + 03
		1.08E + 02	5.19E + 01	4.74E + 01	8.82E + 01

* SE—standard error, **mean value from the whole embryo, all data are presented in relative units

**Fig. 1:**
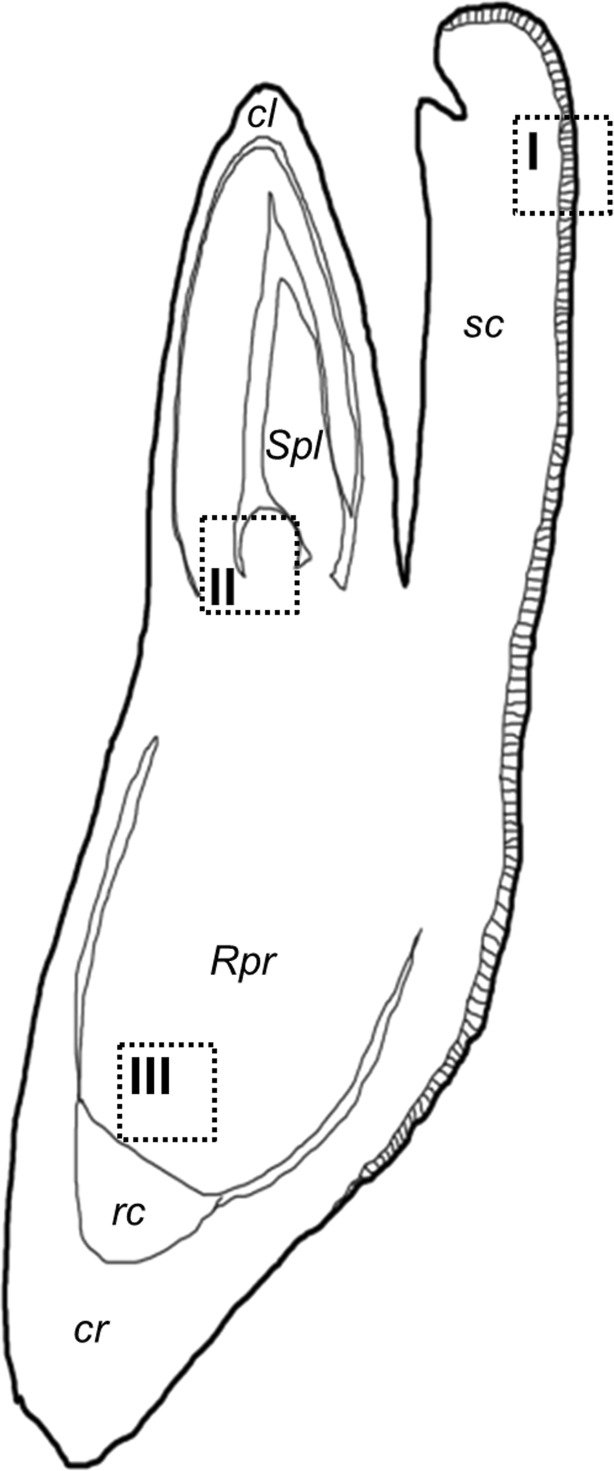
Schematic representation of a longitudinal cross-section through a Brachypodium embryo. *Squares* within the embryo diagram indicate fragments of scutellum (*I*), SAM and leaf primordia (*II*) and RAM (*III*). Enlargements of these areas are shown in Figs. 3, 4, and 5. *cl* coleoptile, *cr* coleorhiza, *rc* root cap, *Rpr* RAM and primary root, *sc* scutellum, *Spl* SAM and primary leaf

^a^Measurements were done from region Rpr of Brachypodium embryo (Fig. 1)

^b^Measurements were done from region Spl of Brachypodium embryo (Fig. 1)

Longitudinal sections were excised from the middle part of each type of embryo and included the scutellum, coleoptile, shoot apical meristem (SAM) with leaf primordia, root apical meristem (RAM), primary root, the root cap and coleorhiza (Fig. 1). For presentation purpose, in the case of Figs. 3, 4, and 5, three representative images from the respective region (I, II and III) that were obtained from the same embryo section were selected for each type of epigenetic modification.

## Results and discussion

The transition of a seed from a dry state to a metabolically active state is one of the developmental switches in plants that require significant changes in both the spatial and temporal patterns of gene expression. Transcriptional reprogramming involves the active modification of the chromatin structure. During the first phase of germination, which is known as imbibition, many processes that are important for a new plant take place, e.g. DNA and mitochondria repair and the synthesis of proteins using extant and new mRNA (Bewley 1997). These processes, especially de novo transcription, are connected with gene activation.

**Fig. 2:**
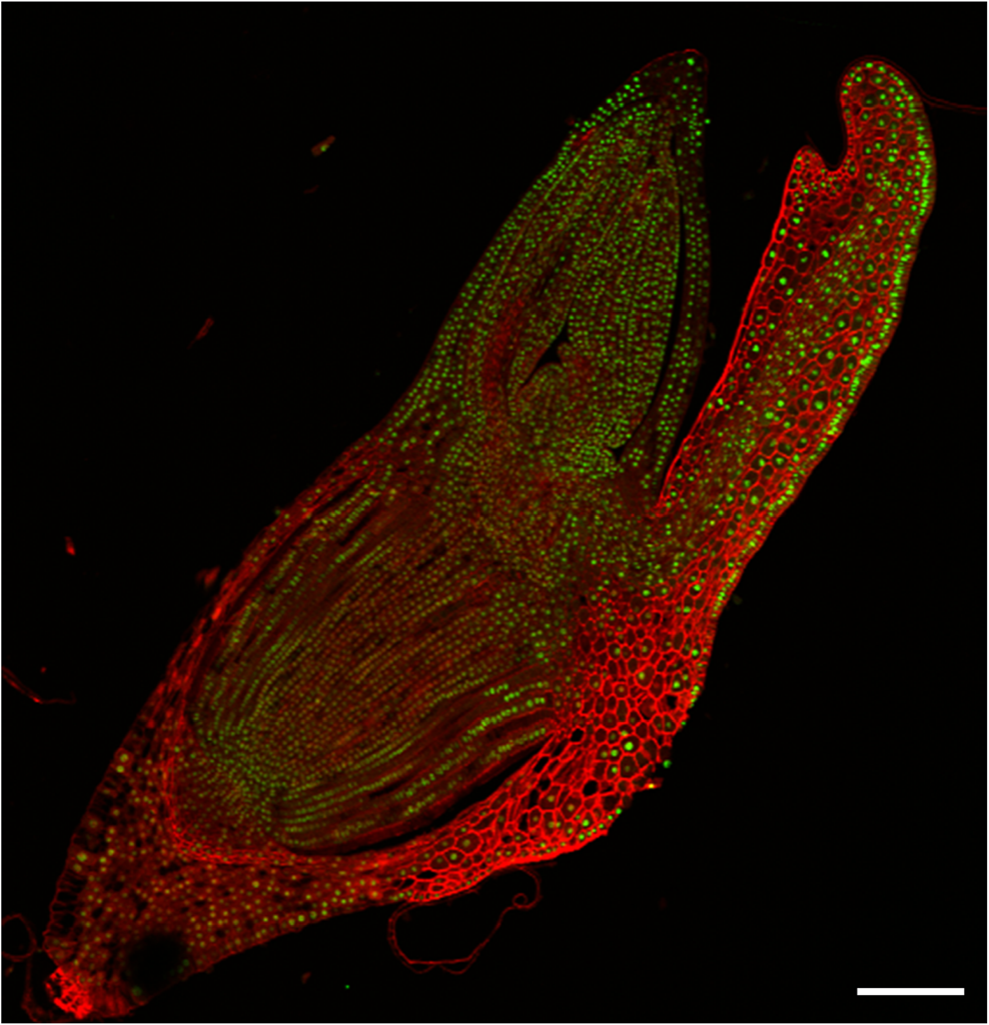
Immunodetection (*green fluorescence*) of unmodified H3 in imbibed Brachypodium embryo. *Red* (*artificial colour*) *fluorescence*—DAPI. *Red colour* of cell walls is caused by their autofluorescence. *Scale bar* represents 50 μm

As a control, immunodetection of the unmodified H3 and H4 was performed for matured, desiccated, imbibed and germinated Brachypodium embryos. The intensity of immunosignals in the nuclei of each embryo type was similar in various organs and tissues (Fig. 2). The results of this measurements indicated that the levels of unmodified histones were rather stable, which was evident in particular for the histone H3 (Table 1, Fig. 2).

### Histone H4 and H3 acetylation (H4K16ac, H3K18ac) considerably increases in embryos during seed imbibition and germination

**Fig. 3:**
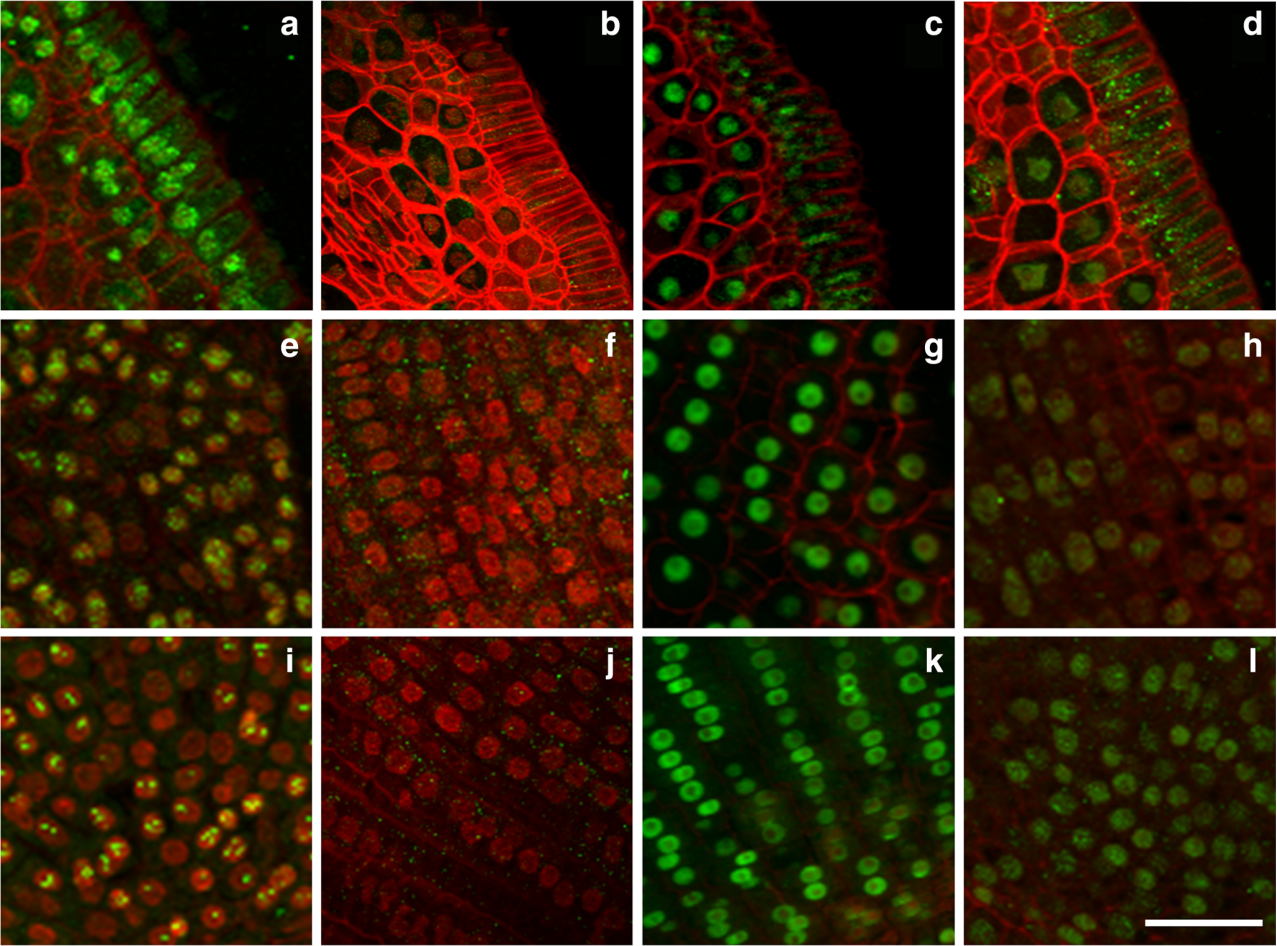
Immunodetection (*green fluorescence*) of H4K16ac in matured (**a**, **e**, **i**), ‘dry’ (**b**, **f**, **j**), imbibed (**c**, **g**, **k**) and germinating (**d**, **h**, **l**) Brachypodium embryos. Fragments of cross-sections through the scutellum (**a**–**d**), SAM (**e**–**h**) and RAM (**i**–**l**). *Red* (*artificial colour*) *fluorescence*—DAPI. *Red colour* of cell walls is caused by their autofluorescence. *Scale bar* represents 20 μm; all photomicrographs were taken at the same magnification

When the seeds progressed from maturation to desiccation and then the imbibition and germination stages, the level of H4K16 acetylation in the scutellum, SAM and RAM of Brachypodium embryos changed (Table 1). This modification was evident in the epithelial cells of the scutellum during seed maturation (Fig. 3a), whereas it was hardly detectable in desiccated seeds (Fig. 3b). In turn, the level of H4K16ac increased significantly in all of the scutellum tissues during seed imbibition (Fig. 3c), whilst it was detectable to the lesser degree in the scutellum, and interestingly, in the cytoplasm of epithelial cells after germination (Fig. 3d). A similar pattern of this modification was detected in desiccated seeds in SAM and RAM, where H4K16ac was almost undetectable in majority of nuclei (Fig. 3f, j), then significantly increased during imbibition (Fig. 3g, k) and decreased at the later stages of germination (Fig. 3h, l). In the SAM and RAM of embryos during seed maturation, the interphase nuclei were characterised by a dot-like pattern of H4K16ac (Fig. 3e, i). At the end of seed maturation, the desiccation process results in the inhibition of DNA replication and the accumulation of nuclei at the G1 and G0 phases of the cell cycle (Deltour 1985; Sliwinska 2009). The strong immunosignals of H4K16ac that were visible in the nuclei of matured Brachypodium embryo cells may then be indicators of the G1 phase (Jasencakova et al. 2000).

**Fig. 4:**
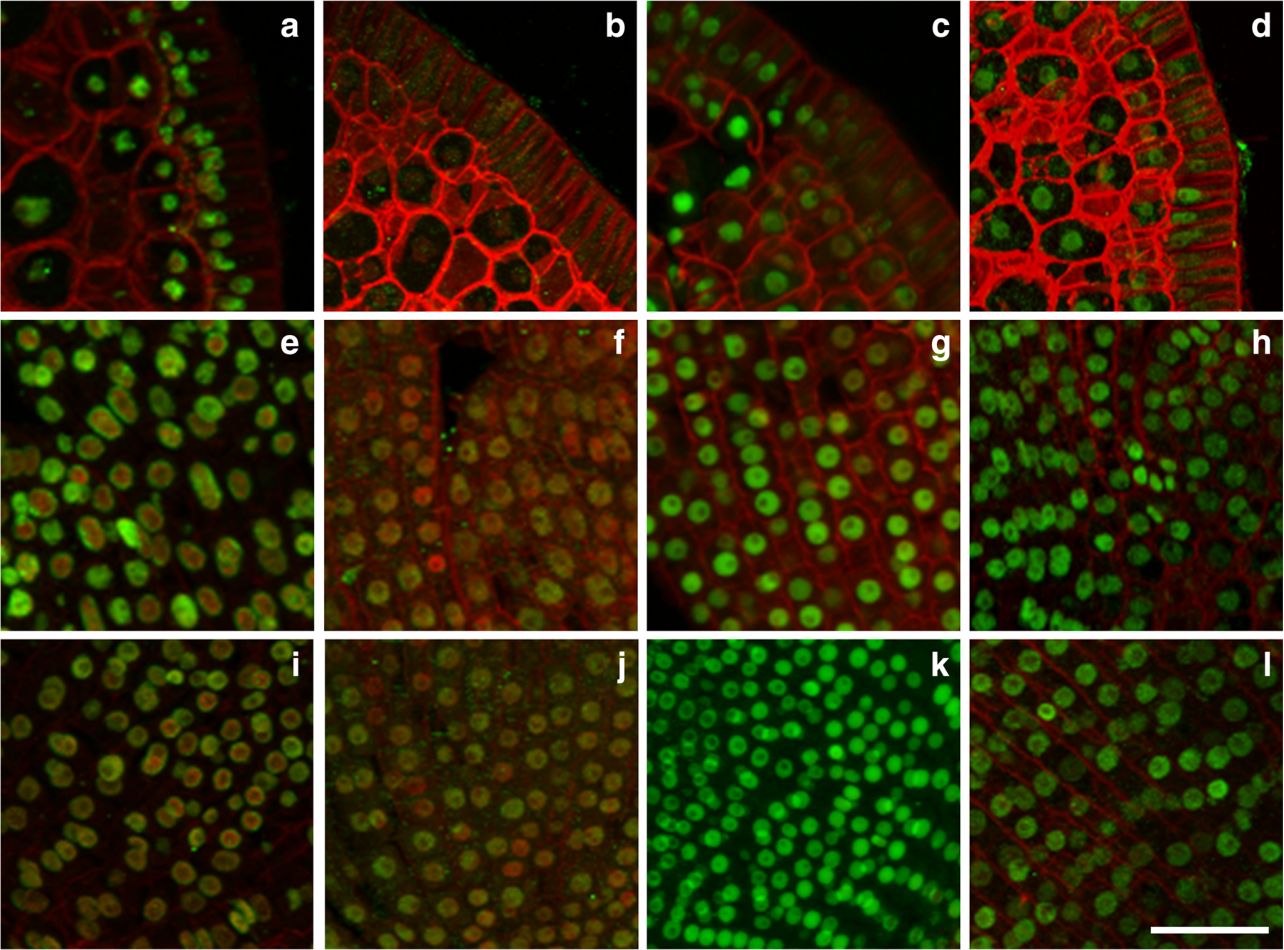
Immunodetection (*green fluorescence*) of H3K18ac in matured (**a**, **e**, **i**), ‘dry’ (**b**, **f**, **j**), imbibed (**c**, **g**, **k**) and germinating (**d**, **h**, **l**) Brachypodium embryos. Fragments of cross-sections through the scutellum (**a**–**d**), SAM (**e**–**h**) and RAM (**i**–**l**). *Red* (*artificial colour*) *fluorescence*—DAPI. *Red colour* of cell walls is caused by their autofluorescence. *Scale bar* represents 20 μm; all photomicrographs were taken at the same magnification

Acetylation of H3K18 and H4K16 is considered as euchromatin marks. The first modification is generally involved in the promotion of transcription, whereas H4K16ac plays an important role during replication (Jasencakova et al. 2000, 2001). Both modifications were detected at the highest level in the imbibed embryos, especially in RAM and SAM (Figs. 3g, k and 4g, k). A high level of H4K16ac in the meristems of imbibed embryos (Fig. 3g, k) indicates that an intensive process of DNA replication may occur at the early germination phase. Acetylation of H4K5 is another modification that is linked with DNA replication. A high level of this modification was also detected in the nuclei of the imbibed embryos (data not shown). However, DNA replication is a relatively late event in germination (Bewley 1997; Sliwinska 2009). In order to validate the appearance of DNA replication during the first 4 h of imbibition, we performed the experiment with EdU incorporation; however, we did not observe any anti-EdU signals in the imbibed embryos. This observation may indicate that high levels of H4 acetylation are linked with a process or processes other than DNA replication. It is possible that the strong H4K16ac and H4K5ac immunosignals in the imbibed Brachypodium embryos reflect the chromatin reorganisation that is connected with transcriptional reprogramming during the developmental switch from an inactive and dry state to an imbibed and metabolically active one. Chromatin reorganisation during developmental transition has been observed in *Arabidopsis thaliana* (Arabidopsis), for example during floral transition (Tessadori et al. 2007b), cellular dedifferentiation (Tessadori et al. 2007a) and seed maturation (van Zanten et al. 2011, 2012). During the early phase of imbibition, there is a change in gene activity that is regulated by the modification of chromatin structure at the epigenetic level. The acetylation of H4 alters histone-DNA interactions and facilitates the access and binding of transcription factors. The high level of these modifications probably reflects the chromatin reorganisation that is involved in redefining the transcription programme during the first phase of germination.

Acetylation of H3K18, which is linked with transcription, was another modification that was investigated. It was high in the scutellum during seed maturation (Fig. 4a), imbibition (Fig. 4c) and germination (Fig. 4d). However, significant differences in the levels of H3 acetylation were found in the epithelial cells of the scutellum. Whilst this modification was evident in all of the epithelial cells in the scutellum during maturation (Fig. 4a), it was only detectable in the distal part of the epithelial cells during imbibition and germination (Fig. 4c, d). H3K18ac was almost undetectable in any scutellum tissue during seed desiccation (Fig. 4b). In SAM and RAM, H3K18ac was the highest during seed imbibition (Fig. 4g, k) and germination (Fig. 4h, l) and the lowest in desiccated seeds (Fig. 4f, j). In matured seeds, this modification produced specific patterns in the interphase nuclei from SAM and RAM, which formed characteristic, ring-shaped patterns at their perimeter in most of them (Fig. 4e, i).

H3K18ac was the highest during seed imbibition and germination and the lowest in desiccated seeds. van Zanten et al. (2014) reported that Arabidopsis deacetylase HDA9 is a negative regulator of germination. *Hda9* mutants have elevated histone acetylation levels, reduced seed dormancy and germinate significantly faster than the wild-type plants. This implies that histone deacetylation plays a role in the transition from seed to seedling (van Zanten et al. 2014) and it is imperative for the repression of genes that affect seed germination positively (Nonogaki 2014). It is possible that the weak H3K18ac immunosignals that are visible in the nuclei of ‘dry’ embryos may indicate that this histone modification can be associated with the transcriptional activity of embryo cells during seed storage. Comai and Harada (1990) concluded that the transcriptional competence of several genes was reduced though not absent in the dry seeds of rapeseed compared with matured ones.

Desiccation is the signal that is required for the ‘switch’ in gene expression programmes from an embryonic to a postgerminative pattern. Analysis of the specific transcripts that are produced in dry seed nuclei indicated that the changes in the gene expression patterns that are associated with germination are not initiated during late embryogenesis. These results suggest that the transition from an embryonic to a postgerminative developmental programme occurs after seeds are rehydrated (Comai and Harada 1990). This may explain the significant differences in the histone modification levels between desiccated and imbibed Brachypodium embryos. The results presented in this paper indicate that the abundance of H3K18ac and H4K16ac modifications differs in the various organs and tissues of the four types of Brachypodium embryos. This corroborates our earlier observations that were linked with the distribution of three other modifications in Brachypodium embryos, i.e. H4K5ac, H3K4me1 and H3K4me2 (Wolny et al. 2014). To conclude, based on the results of the measurements of immunosignal intensities, the level of acetylated histones H3K18 and H4K16 was the highest in the imbibed embryos, slightly lower and similar in the matured and germinating embryos, whereas it was considerably lower in the ‘dry’ embryos (Table 1).

### DNA methylation is considerably more stable in embryos during seed development

**Fig. 5:**
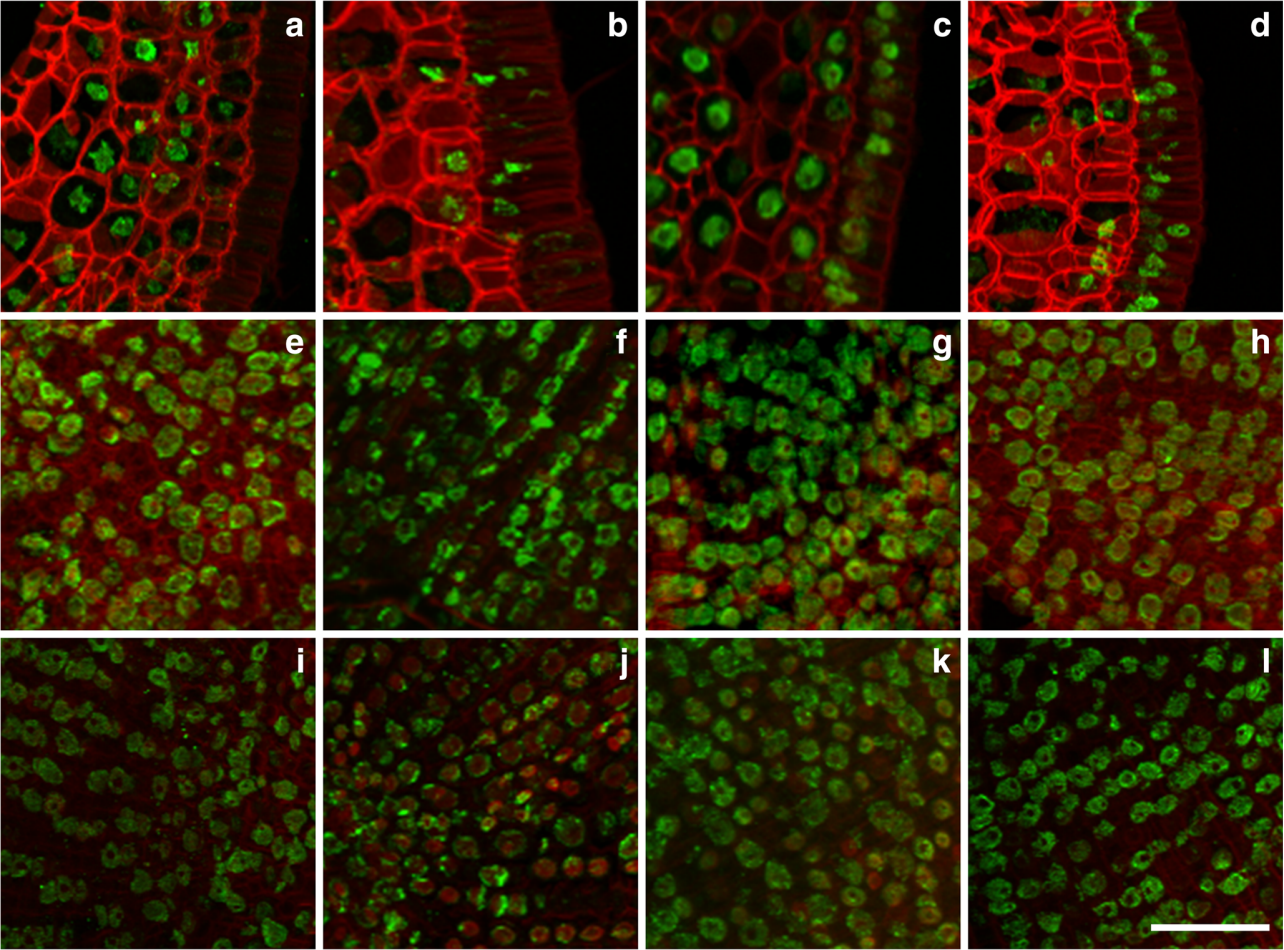
Immunodetection (*green fluorescence*) of 5mC in matured (**a**, **e**, **i**), ‘dry’ (**b**, **f**, **j**), imbibed (**c**, **g**, **k**) and germinating (**d**, **h**, **l**) Brachypodium embryos. Fragments of cross-sections through the scutellum (**a**–**d**), SAM (**e**–**h**) and RAM (**i**–**l**). *Red* (*artificial colour*) *fluorescence*—DAPI. *Red colour* of cell walls is caused by their autofluorescence. *Scale bar* represents 20 μm; all photomicrographs were taken at the same magnification

DNA methylation was another epigenetic modification that was studied in the Brachypodium embryos. Generally, the immunosignal intensity level of 5mC was similar in each type of analysed embryo (Table 1) and was uniformly distributed in all of the analysed tissues and organs; however, some subtle differences were observed in the immunostaining during seed development (Fig. 5). In the scutellum, 5mC was the most abundant in the embryos of imbibed (Fig. 5c) and germinating (Fig. 5d) seeds, whilst it was less abundant in the desiccated ones (Fig. 3b). On the other hand, during seed maturation, 5mC was mainly found in the scutellum provascular cells and in the distal part of scutellum (Fig. 5a). In SAM, this modification was the most abundant during seed maturation and desiccation (Fig. 5e, f), whereas it was less abundant in RAM during desiccation (Fig. 5j). In contrast to the epigenetic modifications that were observed in the histones, global levels of DNA methylation did not display any apparent tissue specificity and were rather uniform among the different types of embryos analysed (Table 1). Genome-wide studies of the DNA methylome in Arabidopsis, rice, maize and soybean (Hsieh et al. 2009; Zemach et al. 2010; Eichten et al. 2013; Song et al. 2013; Xing et al. 2015) have provided important information on the function of DNA methylation in gene regulation during seed development. In plants, specific cell types or tissues, which are associated with reproduction, exhibit altered DNA methylation levels. A good example is the genome-wide DNA demethylation that was observed in the Arabidopsis endosperm (Hsieh et al. 2009) or the local DNA hypomethylation that activates genes in the rice endosperm (Zemach et al. 2010). Recently, Xing et al. (2015) characterised the dynamic DNA methylation profiles across rice seed development and revealed that the embryo is primarily hypermethylated around the non-transposable element (TE) genes, short DNA-TEs and short interspersed TEs as compared to the endosperm. It has been suggested that these genes, whose expression is controlled by DNA methylation, may be important for the regulation of seed development.
